# Obstructive sleep apnoea and hypertension: Current understanding, diagnosis and management

**DOI:** 10.1016/j.clinme.2026.100575

**Published:** 2026-03-26

**Authors:** Medhia Afzal, Alana Livesey, Mohammed Awais Hameed, Rahul Mukherjee

**Affiliations:** a University Hospital Birmingham NHS Foundation Trust - Heartlands Hospital, Bordesley Green East, Birmingham B9 5SS, United Kingdom; b University of Birmingham, Edgbaston, Birmingham B15 2TT, United Kingdom

**Keywords:** Obstructive sleep apnoea, Hypertension, Resistant Hypertension, CPAP

## Abstract

Obstructive sleep apnoea (OSA) is a common, underdiagnosed disorder characterised by upper airway collapse, intermittent hypoxia and sympathetic activation. Hypertension frequently coexists with OSA, with evidence supporting OSA-driven hypertension mediated by sympathetic overactivity, RAAS (renin–angiotensin–aldosterone system) activation and endothelial dysfunction. OSA-related hypertension often manifests as nocturnal hypertension and is linked to adverse cardiovascular outcomes. Prevalence is high in resistant hypertension, supporting targeted screening. Diagnosis should be considered in patients with daytime somnolence or refractory hypertension, using validated screening tools and sleep studies. Continuous positive airway pressure (CPAP) is the cornerstone of OSA management and can modestly lower blood pressure with cardiovascular benefit. Pharmacological management should follow hypertension guidelines, favouring agents that target relevant mechanisms (eg RAAS blockade and beta-blockade). Aldosterone antagonists are recommended in resistant hypertension. Emerging therapies, including endothelin receptor antagonists and GLP-1 receptor agonists, show promise but require further trials. This article reviews the epidemiology, mechanisms, diagnosis and management of OSA-related hypertension.

## Introduction

Obstructive sleep apnoea (OSA) is characterised by recurrent upper airway collapse during sleep due to reduced pharyngeal tone. This causes apnoeas or hypopnoeas, leading to intermittent hypoxaemia, sleep fragmentation and sympathetic activation.^1^ OSA is defined as an apnoea-hypopnoea index (AHI) ≥15 events/h in asymptomatic patients or ≥5 with symptoms.^2^ Moderate-to-severe OSA affects approximately 5% of adults aged 30–70, yet only 1.4% of adults in the UK have a recorded diagnosis.^3^ OSA is increasingly recognised as a major public health concern due to its strong association with cardiometabolic disease, particularly hypertension.

Hypertension is a leading preventable risk factor for cardiovascular disease and all-cause mortality, with uncontrolled blood pressure (BP) causing over 10 million deaths globally each year.^4^ It affects ∼30% of adults in the UK, with nearly one-third undiagnosed.^5^ Resistant hypertension, defined as elevated BP despite three anti-hypertensive medications at maximally tolerated doses, is strongly associated with OSA, making screening essential in this group.^6^

## Relationship, epidemiology, and clinical significance

OSA and hypertension frequently coexist; over 50% of patients with OSA have hypertension, while 38–56% of hypertensive individuals have OSA.^2^ OSA prevalence is higher in resistant hypertension, affecting up to 80% of patients.^7^ Evidence supports OSA as a contributor to the development and progression of hypertension; however, the reverse causal relationship is less well established.^6^, ^8^ Meta-analyses demonstrate a dose–response association, with greater OSA severity predicting incident hypertension, independent of confounders.^7^, ^9^ Guidelines recognise OSA as a potentially reversible cause of hypertension,^10^ highlighting the importance of early identification and integrated management to reduce cardiovascular risk.

## Pathophysiology: mechanisms linking OSA and hypertension

OSA drives hypertension through repetitive apnoeic events, causing intermittent hypoxia and arousals. Hypoxia stimulates chemoreceptors, increasing sympathetic outflow, leading to transient, then sustained, BP elevation.^7^ Obstructive events generate negative intrathoracic pressure, increasing cardiac afterload and impairing baroreflex regulation. Sleep fragmentation and recurrent arousals stimulate the hypothalamic–pituitary–adrenal axis, elevating catecholamines and cortisol, which may promote metabolic dysfunction.^8^

Sustained sympathetic overactivity activates the renin–angiotensin–aldosterone system (RAAS), promoting fluid retention and contributing to vascular remodelling. Recurrent hypoxia–reoxygenation induces oxidative stress and systemic inflammation, leading to endothelial dysfunction and impaired vasodilation. Endothelin-1 upregulation further drives vasoconstriction and remodelling.

These mechanisms sustain hypertension and increase cardiovascular risk, while metabolic dysfunction and fluid retention may increase OSA severity.^7^, ^8^, ^11^

## Clinical characteristics of hypertension in OSA

Hypertension in OSA is predominantly nocturnal and frequently demonstrates a non-dipping pattern, defined as a <10% fall in nocturnal BP compared with daytime values. Reverse dipping, in which BP rises overnight, may also occur.^2^ Nocturnal hypertension is associated with target organ damage and cardiovascular morbidity, and its prevalence and severity increase with worsening OSA.^1^ The strong link with resistant hypertension supports routine OSA screening in this high-risk population.

### Diagnosis^12^

OSA should be suspected in patients with daytime somnolence, poor sleep quality, loud snoring or uncontrolled hypertension.^13^ Screening tools include the Epworth Sleepiness Scale and STOP-BANG. Epworth assesses subjective daytime sleepiness but lacks diagnostic accuracy, as many OSA patients are not excessively sleepy. STOP-BANG incorporates more parameters, offering high sensitivity but low specificity, making it useful for identifying patients for further investigation.^12^

Definitive diagnosis requires physiological sleep measurements. Methods include overnight oximetry, limited respiratory polygraphy (airflow, oximetry, pulse and thoraco-abdominal movement), and full polysomnography (PSG), which records electroencephalography, electrooculography and electromyography in a sleep laboratory. NICE recommends home respiratory polygraphy as first line for suspected OSA. Where unavailable, oximetry may be used, but is less reliable. If suspicion persists after negative polygraph or oximetry, PSG is recommended.

Home polygraphy is more cost-effective than hospital-based studies and more accurate than oximetry, though oximetry remains common due to limited equipment. PSG is resource-intensive and costly, driving the uptake of newer home-based diagnostic devices (eg AcuPebble, WatchPAT) that enable postal delivery and return. These improve access for patients with mobility or geographic barriers, but may be unsuitable for those with cognitive or dexterity limitations. This technology offers a cost-effective way to expand access and reduce wait times, facilitating earlier diagnosis and management of OSA and hypertension.

NICE recommends ambulatory BP monitoring (ABPM) to confirm hypertension when clinic BP ≥140/90.^10^ ABPM provides detailed analysis including non-dipping and nocturnal hypertension, patterns strongly associated with OSA.^2^

## Treatment

### Lifestyle modification

Weight loss, smoking cessation and optimising sleep hygiene should be advised in OSA and hypertension. Weight reduction through diet, exercise or bariatric surgery lowers BP and improves OSA severity.^6^

### Pharmacological

Current hypertension guidelines do not provide specific recommendations for patients with coexisting OSA and hypertension. Given the increased sympathetic drive and RAAS activation in OSA, agents that block these pathways, eg ACE inhibitors, angiotensin receptor blockers and beta-blockers, may be beneficial. However, evidence to support this is limited to small comparative studies.^7^

Aldosterone antagonists (spironolactone and eplerenone) significantly reduce BP in OSA and may reduce apnoeic events by decreasing laryngeal oedema.^7^ Secondary aldosteronism may be more common in patients with moderate to severe OSA, partly explaining this response. NICE recommends them as a fourth-line therapy in patients with resistant hypertension, yet emerging data suggest that earlier use may be beneficial in OSA-related hypertension; however, larger studies are required.^10^, ^14^ Efficacy may be limited by the ‘aldosterone phenomenon’, in which aldosterone production resumes despite RAAS blockade. Novel selective aldosterone synthase inhibitors (ASIs), including lorundrostat and baxdrostat, show promising BP reduction and may better target hyperaldosteronism seen in OSA, but OSA-specific trials are lacking.^15^

For resistant hypertension, NICE recommends assessing adherence before escalating treatment.^10^ Toxicology screening and directly observed therapy indicate that one-third of apparent resistant cases are due to non-adherence.^16^ The reasons are multifactorial, but a higher pill burden is consistently associated. Single pill combinations improve adherence and BP control compared with separate tablets, although use remains limited by commercial availability.^16^, ^17^ Long-acting RNA-based therapies (zilebesiran) inhibit hepatic angiotensinogen synthesis and can provide sustained BP reduction with biannual dosing. They may improve adherence, but again, OSA-specific trials are lacking.^1^

Aprocitentan, an endothelin receptor antagonist, is approved in the UK for resistant hypertension. Given endothelin-mediated vasoconstriction and vascular remodelling in OSA, this represents a promising option for OSA-related hypertension, although direct trials in this population are lacking.^18^

Sodium-glucose-cotransporter-2 inhibitors reduce cardiovascular events, and small studies in OSA with cardiometabolic comorbidities suggest improvement in AHI, nocturnal oxygenation and nocturnal BP.^1^ Canagliflozin demonstrated nocturnal BP reduction in resistant hypertension and OSA^14^; however, robust trials are needed.

Glucagon-like peptide-1 receptor agonists (GLP-1RAs) can reduce OSA severity and lower BP; a meta-analysis found improved AHI and mean BP reductions of 4.81 mmHg systolic and 0.32 mmHg diastolic.^11^ Although benefits are largely weight loss mediated, improvements seen in non-obese patients suggest additional mechanisms such as enhanced respiratory drive reducing airway collapse and anti-inflammatory/antioxidant effects. Newer incretin-based drugs, including triple agonists and oral agents (orforglipron), are under investigation in OSA-focused trials; if weight-independent OSA and BP benefits are confirmed, they may become targeted adjuncts to continuous positive airway pressure (CPAP).^19^

### Continuous positive airway pressure (CPAP)

CPAP is the gold standard for OSA management. A meta-analysis of 68 trials on CPAP reported systolic reductions of 2–3 mmHg and diastolic reductions of 1.5–2 mmHg in patients with OSA. Although modest, this reduction is clinically meaningful, lowering coronary artery disease risk by 7% and stroke risk by 10%.^2^ Greater reductions occur in resistant hypertension (≈6 mmHg systolic, 4 mmHg diastolic)^7^ and in patients with severe nocturnal hypoxaemia.^2^

Combined CPAP and anti-hypertensive therapy achieves superior BP control compared with either alone. Benefits are most pronounced in patients <60 years, with high baseline BP, resistant hypertension or severe OSA.^2^ Optimal benefit requires ≥5 h of use nightly; however, adherence is poor, with 63% of patients non-adherent.^1^, ^12^ CPAP does not address all mechanisms driving hypertension, such as volume overload or aldosterone excess, limiting its role as a standalone therapy.^2^

NICE^12^ recommends fixed-rate CPAP for symptomatic mild OSA and for moderate-to-severe cases, with telemonitoring for at least 12 months.^12^ Telemonitoring facilitates remote optimisation and, when combined with home devices, may reduce health inequalities by widening access for those with limited mobility or geographic barriers.^8^

Despite modest BP effects and adherence challenges, CPAP remains central to OSA care. Current evidence is limited by short follow-up and variable compliance; further research should define CPAP’s role within anti-hypertensive algorithms.

## Current understanding and clinical implications

Although BP reduction with CPAP is modest, even small decreases provide meaningful cardiovascular benefit at a population level. The association between OSA severity and hypertension supports integrated care. Patients with resistant, poorly controlled or non-dipping hypertension should be assessed for OSA, as earlier diagnosis improves BP control and reduces mortality.^1^, ^6^ Expanding access to home-based sleep testing and telemonitoring can shorten diagnostic delays and promote equitable care.^12^

Current guidelines provide limited direction on optimal pharmacotherapy for OSA-related hypertension, highlighting the need for adequately powered trials. Emerging therapies such as endothelin antagonists, ASIs and GLP-1RAs may enable mechanism-based pharmacological strategies alongside CPAP.

CPAP should be implemented across all stages of OSA in hypertensive patients, particularly those with drug-resistant disease.^7^ Earlier identification and treatment of OSA remain central to improving cardiovascular outcomes and reducing the healthcare burden of hypertension.

## Take-home messages

• Obstructive sleep apnoea (OSA) and hypertension frequently coexist. OSA is a recognised contributor to the development and progression of hypertension.
• Resistant or poorly controlled hypertension should prompt routine assessment for underlying obstructive sleep apnoea.
• Nocturnal blood pressure patterns, particularly non-dipping and reverse dipping, are common in obstructive sleep apnoea and are strongly associated with target organ damage and adverse cardiovascular outcomes.
• Pharmacological management should follow standard hypertension guidelines; however, OSA-related mechanisms (sympathetic activation/RAAS upregulation) provide a rationale for ACE inhibitors/ARBs and beta-blockers despite limited evidence. In resistant hypertension, NICE recommends an aldosterone antagonist as fourth-line therapy.
• Continuous positive airway pressure (CPAP) remains central to OSA management. Although blood pressure reductions are modest, CPAP reduces cardiovascular risk and should be used as an adjunct to anti-hypertensive therapy.

## CRediT authorship contribution statement

**Alana Livesey:** Writing – original draft, Conceptualization. **Mohammed Awais Hameed:** Supervision, Project administration, Conceptualization. **Medhia Afzal:** Writing – review & editing, Writing – original draft, Conceptualization. **Rahul Mukherjee:** Supervision, Project administration, Conceptualization.

## Funding

This article did not receive any specific grant from funding agencies in the public, commercial or not-for-profit sectors.

## Declaration of competing interest

The authors declare that they have no known competing financial interests or personal relationships that could have appeared to influence the work reported in this paper.
